# Supplementary material for: Use of Intravaginal Progesterone-Releasing Device Results in Similar Pregnancy Rates and Losses to Long-Acting Progesterone to Synchronize Acyclic Embryo Recipient Mares
Source: Vet Sci. 2021 Sep 10;8(9):190. doi: 10.3390/vetsci8090190 (PMC8472191; doi:10.3390/vetsci8090190)
Supplement: Supplementary file 1 [file vetsci-08-00190-s001.zip › vetsci-1330954-SI.pdf]

veterinary sciences

MDPI

Article

Use of Intravaginal Progesterone-Releasing Device Results in Similar Pregnancy Rates and Losses to Long-Acting Progesterone to Synchronize Acyclic Embryo Recipient Mares

Lorenzo G .T. M. Segabinazzi [1,2,†], Luiz R. P. Andrade Jr. [1], Marco A. Alvarenga [1], Jose A. Dell'Aqua Jr. [1] and Igor F. Canisso [2,*]

Supplementary

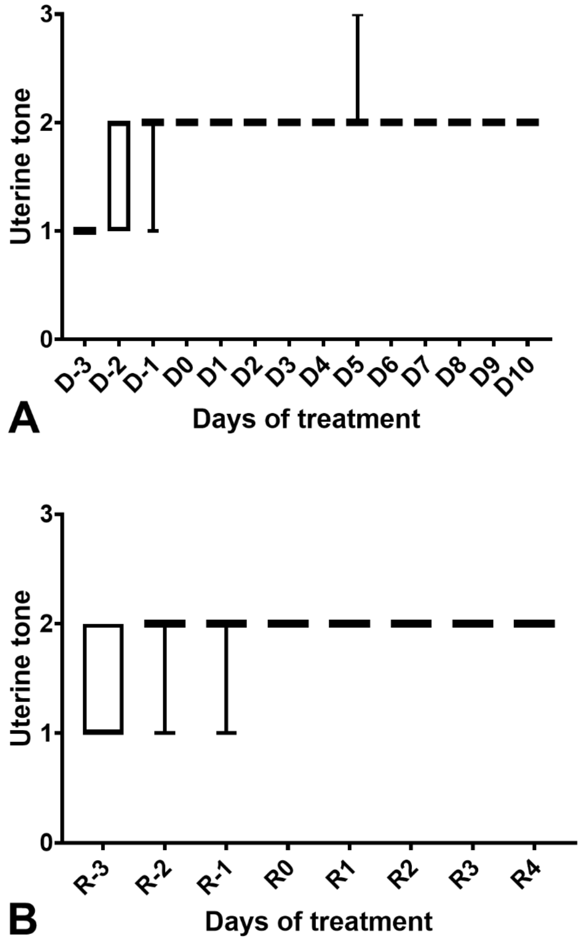

Figure S1. Median and interquartile ranges of uterine tone of acyclic mares (n = 12). (**A**) Mares were treated with estradiol-17β for 3 days (D-3 to D-1) and then given an intravaginal progesterone releasing device (IPRD, D0). The IPRD was kept for nine days and was then removed (D9). (**B**) Then, the mares were resynchronized three days post-removal of IPRD after the first synchronization. Mares were again treated with estradiol-17β for 3 days (R-3 to R-1) and then had a new IPRD inserted (R0). The IPRD was removed three days (R3) later. Score of 1 represents a flaccid and floppy uterine tone (consistent with anestrus), whereas a score of 3 represents a well-toned, rounded, tubular uterus.
